# Effect Estimates, Effect Sizes, Confidence Intervals, and P-values: A Framework for Reporting and Interpreting Biomedical Research

**DOI:** 10.7759/cureus.116428

**Published:** 2026-09-17

**Authors:** Himel Mondal, Sukrita Sukrita, Anjali Sinha, Hyma Jini, Pradosh Kumar Sarangi

**Affiliations:** 1 Physiology, All India Institute of Medical Sciences, Deoghar, IND; 2 Radiodiagnosis, All India Institute of Medical Sciences, Deoghar, IND

**Keywords:** biomedical statistics, clinical significance, cohen’s d, confidence interval, effect estimate, effect size, p-value, r shiny application, statistical interpretation, statistical significance

## Abstract

The interpretation of biomedical research findings should extend beyond statistical significance. Effect estimates, effect sizes, confidence intervals, and P-values provide information regarding the magnitude, direction, precision, and statistical evidence of an observed finding. However, these concepts are frequently misunderstood or interpreted in isolation, potentially leading to inappropriate conclusions about their clinical importance. This article presents a framework comprising effect estimate, effect size, confidence interval, and P-value (E-E-C-P) to facilitate comprehensive interpretation of biomedical results. Using clinically relevant examples, the framework demonstrates how these measures address different but interconnected questions: what was observed, how large the effect was, how precise the estimate is, and how strong the statistical evidence is. To translate these concepts into an interactive learning environment, an R Shiny application (University of Auckland in Auckland, New Zealand), EffectSight, was developed as a pedagogical tool using a single illustrative example focused on Cohen’s d. The application allows learners to modify group means, standard deviations, and sample sizes and observe how these changes influence the effect estimate, Cohen’s d, confidence interval, and P-value, with support from graphical visualizations and the E-E-C-P analysis grid. The application was designed specifically to facilitate conceptual understanding rather than to serve as a comprehensive statistical analysis tool. The E-E-C-P framework emphasizes that statistical significance should be interpreted alongside the magnitude and precision of an observed effect rather than considered in isolation.

## Introduction

Statistical analysis is fundamental to biomedical research because it enables investigators to quantify the effects of interventions, evaluate associations, and draw conclusions from sample data [1]. The P-value has been the most frequently reported statistical measure for determining whether an observed result is statistically significant. However, reliance on the P-value alone has been widely criticized because it provides limited information about the magnitude, direction, and precision of an observed effect [2]. A statistically significant result does not necessarily indicate a clinically meaningful difference, while a nonsignificant result does not always imply the absence of an important effect [3].

To facilitate more informative interpretation of research findings, it is recommended to report the effect estimate and/or effect size and confidence interval along with the P-value [4]. Despite these recommendations, many early-career researchers, postgraduate students, and healthcare professionals continue to rely primarily on P-values when interpreting study findings [5]. Confusion between concepts such as effect estimate and effect size, as well as misunderstandings regarding the interpretation of confidence intervals, remains common. These misconceptions may lead to inappropriate conclusions regarding the importance, reliability, or applicability of research findings.

With this background, this article aims to provide a practical framework for understanding the complementary roles of effect estimates, effect sizes, confidence intervals, and P-values in biomedical research. This framework should be considered a general inferential framework for better reporting of research findings.

## Technical report

A clinically oriented framework

Brief definitions of effect estimate, effect size, confidence interval, and P-value (E-E-C-P) are presented in Table 1. In this context, the term “effect measure” is frequently encountered. An effect measure describes the statistical metric used to quantify an effect (e.g., mean difference or standardized mean difference) [6]. An effect estimate is the numerical value that quantifies the observed effect in a study. Effect size expresses the magnitude of an effect [7,8]. A 95% confidence interval is a range of values calculated from a sample to estimate an unknown, fixed population mean [9]. The P-value indicates how compatible the observed data are with the null hypothesis. A smaller P-value indicates that the observed result would be less likely if the null hypothesis were true, providing stronger evidence against the null hypothesis [10]. 

**Table 1:** Brief of effect estimates, effect sizes, confidence intervals, and P-values *A Cohen’s d of 0.80 is conventionally classified as a large standardized effect according to Cohen’s heuristic thresholds; these thresholds are not universal and should be interpreted in context [8]. BP: blood pressure, CI: confidence interval, mmHg: millimeters of mercury, E: effect estimate/effect size, C: confidence interval, P: P-value. This table was created by the authors by synthesizing the literature [6-10].

Step	Statistical concept	Simple question	Biomedical example	Interpretation
E	Effect estimate	What is the observed effect?	Mean difference in systolic BP = −8 mmHg	The antihypertensive drug reduced systolic BP by an average of 8 mmHg compared with placebo
E	Effect size	How large is the effect?	Cohen's d = 0.80	The difference corresponds to 0.80 standard deviations, indicating a large effect*
C	Confidence interval	How precise is the estimate?	95% CI = −12 to −4 mmHg	The data are compatible with a true average difference ranging from a reduction of 4 to 12 mmHg
P	P-value	How strong is the evidence against the null hypothesis?	p = 0.003	If the drug truly had no effect, a difference this large or larger would be observed in about 3 out of 1,000 similar studies

As many novice researchers have misconceptions about the interpretation of 95% confidence intervals, we present a visual summary (Figure 1) to facilitate understanding. To estimate the mean systolic blood pressure (BP) of 5,000 medical students (the population), researchers may select 100 students (the sample) and get a mean systolic BP from the sample (122 mmHg), and calculate a 95% confidence interval of 118-126 mmHg. They conclude that they are 95% confident the true population mean (e.g., 120 mmHg) lies between 118 and 126 mmHg. To clarify what this "95% confident" truly means, imagine that researchers repeatedly draw fresh samples of 100 students from that same population and compute a new confidence interval each time. If they repeat this study 100 times, approximately 95 of those 100 intervals will successfully contain the true population mean (120 mmHg), while about 5 will miss it by chance. 

**Figure 1:**
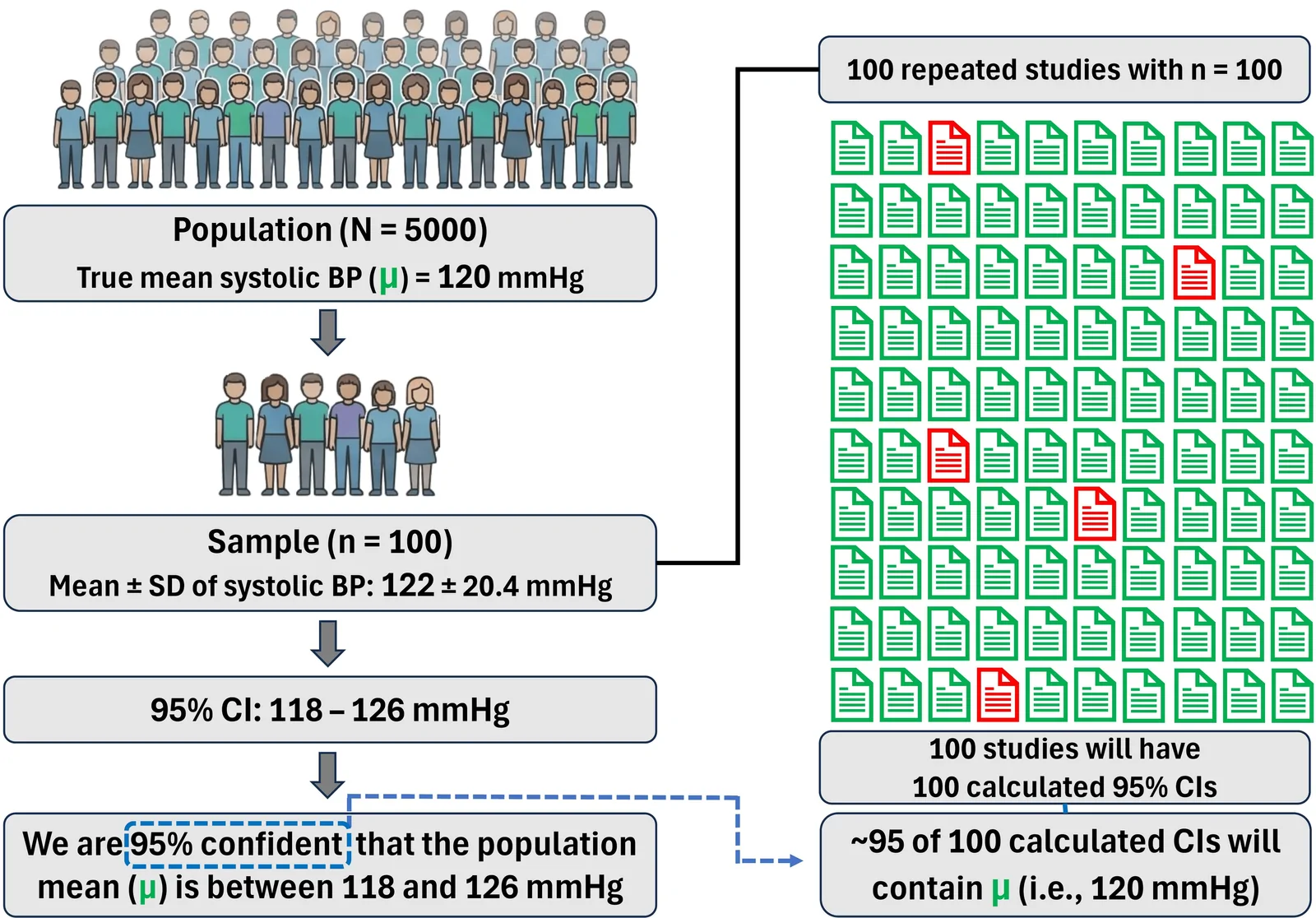
An example for explaining the 95% confidence interval If we repeatedly take 100 random samples (100 studies) of 100 people (the same number as the primary study) and calculate a 95% CI each time, approximately 95 of the 100 CIs will contain the true population mean (μ = 120 mmHg). The figure was created by Dr. Himel Mondal using Sketchbook (Sketchbook, Inc., Toronto, Canada) and PowerPoint (Microsoft Corporation, Redmond, Washington).

Here, we propose a framework, shown in Figure 2. The E-E-C-P pyramid represents a progressive approach to interpreting biomedical findings. The broad base begins with the effect estimate, which provides the empirical foundation of interpretation. Subsequent levels add information about magnitude and precision, while the apex addresses statistical evidence through the P-value. The decreasing width of the pyramid represents progressive refinement of interpretation rather than decreasing statistical importance. The P-value therefore occupies the apex because it is considered after the effect, its magnitude, and its precision have been examined, rather than because it is the least important measure.

**Figure 2:**
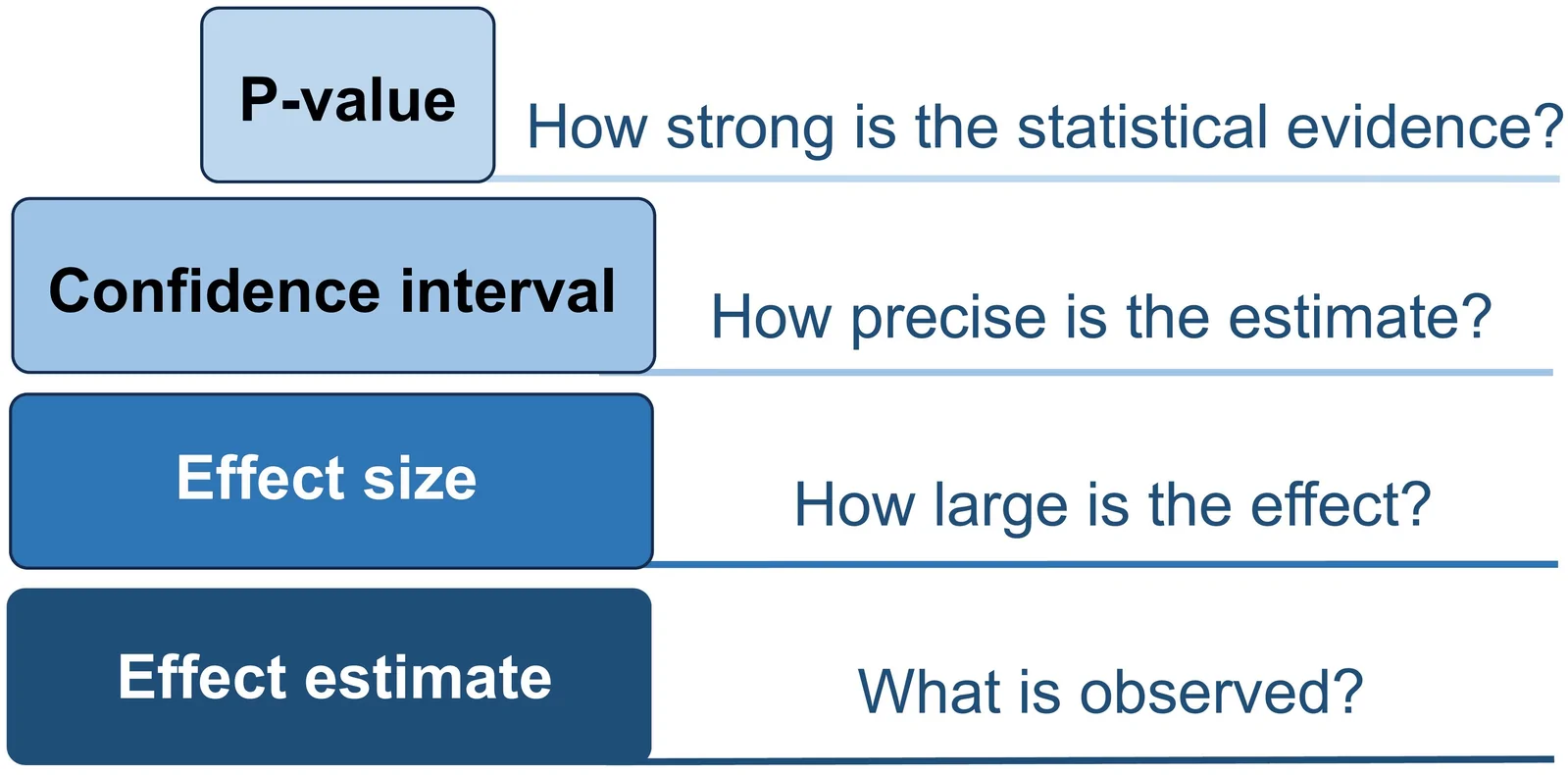
The E–E–C–P framework for reporting and interpretation of biomedical research findings It illustrates an approach to interpreting research results, beginning with the effect estimate as the empirical foundation and progressing through effect size, confidence interval (CI), and P-value (P). Each level addresses a distinct but interconnected question: What was observed? How large is the effect? How precise is the estimate? What is the strength of the statistical evidence against the null hypothesis? This figure was created by Dr. Himel Mondal.

All four can contribute to the interpretation of a biomedical result. Taken together, these measures provide a more complete picture than any single statistical value. The emphasis should therefore move beyond simply asking whether a result is statistically significant. A meaningful interpretation should consider the size of the effect, its precision, the statistical evidence, and its clinical relevance.

Common effect estimate and size

Biomedical research uses several measures to describe the magnitude of an observed effect. The choice depends on the type of outcome and the research question [11]. Common non-standardized effect estimates include mean difference, risk difference, risk ratio, and odds ratio, which retain the original units or have a direct clinical interpretation. Standardized effect sizes, such as Cohen’s d, express an effect relative to the variability of the measurements and are particularly useful when the same outcome is assessed using different scales [12]. Understanding these measures helps researchers interpret not only whether an effect exists, but also how large the effect is and what it means in clinical or practical terms. Common effect estimates and sizes are shown in Table 2 [7,13,14].

**Table 2:** Common effect sizes used in biomedical research ANOVA: analysis of variance, SD: standard deviation, RCT: randomized controlled trial. The table was created by the authors by synthesizing the literature [7,13,14].

Domain or situation	Standardized/unit-free effect-size measure	Symbol	What it represents	Typical use
Two-group continuous outcome	Cohen's standardized mean difference	d	Difference between two means expressed in SD units, commonly using the pooled SD	RCTs, experimental studies
Two-group continuous outcome	Hedges' standardized mean difference	g	Small-sample bias-corrected standardized mean difference	Small samples, meta-analysis
Two-group continuous outcome	Glass's standardized mean difference	Δ	Mean difference divided by the SD of a reference group, usually the control group	When treatment may affect variability
Correlation or association	Pearson correlation coefficient	r	Strength and direction of a linear association	Correlation studies
Correlation or association	Spearman rank correlation coefficient	ρ or *r*_s_	Strength and direction of a monotonic association	Ordinal or non-normal data
Binary × continuous variables	Point-biserial correlation coefficient	r_pb_	Association between a binary variable and a continuous variable	Binary group × continuous outcome
Regression or prediction	Standardized regression coefficient	β	Change in outcome, in SD units, associated with a 1-SD change in the predictor, conditional on other predictors	Multiple regression
Association after adjustment	Partial correlation	r_p_	Association between two variables after controlling for other variables	Adjusted association
Regression	Semipartial correlation	sr	Unique association between a predictor and outcome after removing the influence of other predictors from the predictor	Multiple regression
ANOVA or multiple groups	Eta squared	η²	Proportion of total outcome variance associated with a factor	ANOVA
ANOVA	Partial eta squared	η_p_^2^	Proportion of variance associated with an effect relative to the effect plus its error variance	Factorial or repeated-measures ANOVA
ANOVA	Omega squared	ω²	Less biased estimate of the population variance associated with an effect	ANOVA
ANOVA or power analysis	Cohen's f	f	Standardized measure of between-group variability relative to within-group variability	ANOVA, power analysis
Categorical association	Phi coefficient	φ	Strength of association between two binary variables	2 × 2 contingency tables
Categorical association	Cramér's V	V	Strength of association between categorical variables	Larger contingency tables

Standardized *effect sizes* are particularly useful when outcomes are measured using different scales or when the magnitude of an effect needs to be compared across studies. In contrast, non-standardized *effect measures*, such as mean differences, retain the original clinical units and are often easier to interpret in biomedical practice [7,12,13]. The term "effect size" is often used inconsistently in the literature and may refer to both standardized and non-standardized effect measures; therefore, the specific measure and its interpretation should always be stated clearly.

Pedagogical Shiny application

To support the pedagogical purpose of this article, we developed an interactive R (University of Auckland in Auckland, New Zealand) Shiny application, EffectSight: Cohen's d [15], to help learners understand effect estimates, effect sizes, confidence intervals, and P-values through an interactive biomedical example. Users can enter the mean, standard deviation, and sample size for two groups and observe how these parameters influence the effect estimate, Cohen's d, confidence interval, and P-value. The application also provides graphical visualization of the two distributions and presents the results using the E-E-C-P analysis grid, allowing learners to explore the concepts by changing the input values rather than relying only on mathematical formulas.

EffectSight was developed as an R Shiny application, with the user interface and reactive computation implemented within a single R-based application structure. This application was developed using R version 4.4.3 (R Foundation for Statistical Computing, Vienna, Austria). The application uses Shiny for interactive user inputs and dynamic output generation, "ggplot2" for graphical visualization of the group distributions, "dplyr" for data manipulation, and shinythemes for the user-interface theme and styling. The statistical calculations are implemented using base R functions, including "qt()" for obtaining t-distribution critical values and "pt()" for calculating the two-sided P-value. The application accepts group means, standard deviations, and sample sizes as inputs and performs the effect estimate, pooled standard deviation, Cohen's d [16], confidence interval, and P-value calculations reactively, with results displayed through the E-E-C-P analysis framework.

How to use the application

The application can be run locally using R and RStudio (RStudio, Posit Software, PBC, Boston) [17,18]. First, users need to install R on the computer, followed by RStudio (Figure 3A) [17]. After opening RStudio, users can copy and paste the EffectSight Shiny application code [15]. The required packages will be installed and loaded as specified in the code. Running the script launches the application in the RStudio viewer or a web browser. Users can enter or modify the treatment and control group means, standard deviations, and sample sizes using the input fields and sliders (Figure 3B). The application automatically updates the graphical distributions and the E-E-C-P analysis grid, allowing learners to observe how changes in the study parameters affect the effect estimate, Cohen's d, confidence interval, and P-value. This interactive approach allows learners to experiment with different scenarios and develop an intuitive understanding of the relationship between effect magnitude, variability, sample size, precision, and statistical significance.

**Figure 3:**
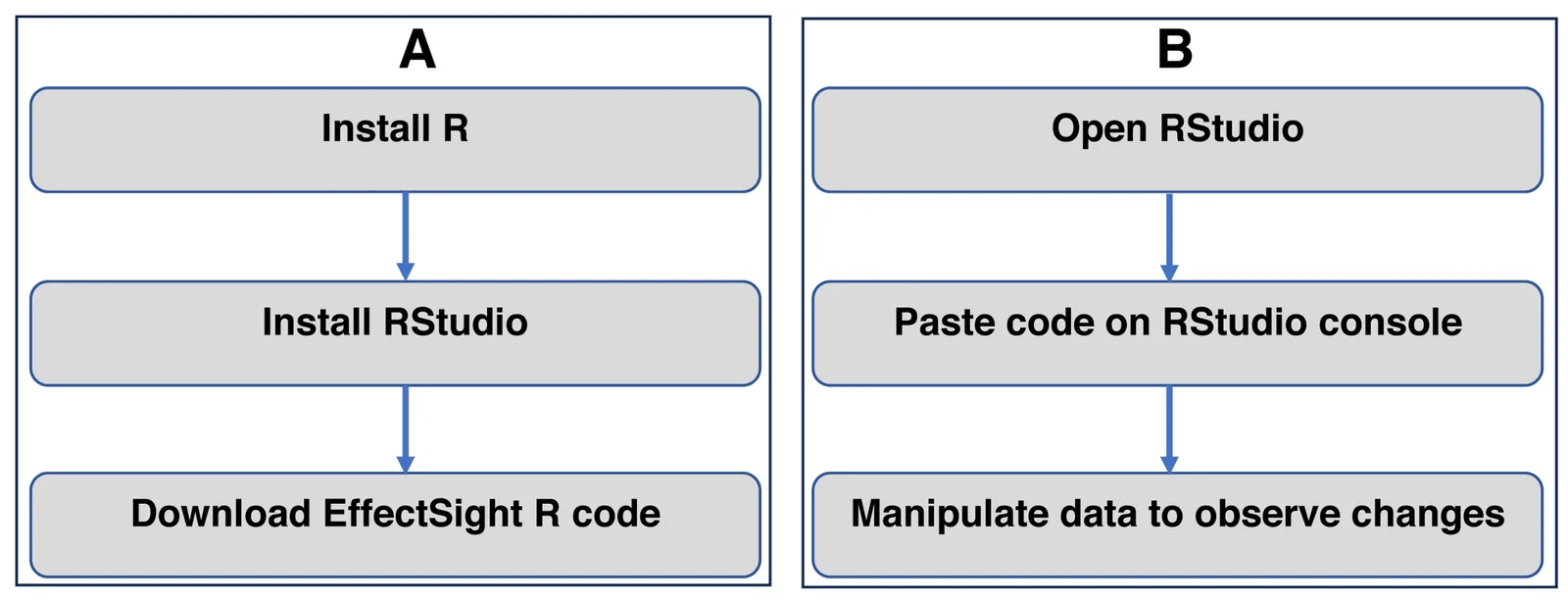
Steps for preparing the computer (A) and opening the application (B)

An example

An example is shown in Figure 4. The population-overlap plot displays the distribution of the two groups and provides a visual representation of the difference between their means. The application then organizes the results into four components of the E-E-C-P analysis grid. The effect estimate presents the raw mean difference in the original clinical measurement and displays the group means and pooled standard deviation. The effect size reports Cohen's d and provides an intuitive interpretation of its magnitude, including the degree of population overlap and common-language interpretation. The confidence interval presents uncertainty around both the raw mean difference and Cohen's d, helping users understand the precision of the estimated effect. Finally, the P-value reports the statistical evidence from the comparison, together with the corresponding t-statistic and degrees of freedom, and indicates whether the result meets the conventional significance threshold. In the example shown, one group has a mean of 132 compared with 130 in the other group, giving a raw mean difference of 2 units, a Cohen's d of approximately 0.19, with a P-value of 0.0466. This example illustrates an important teaching point of the application: a result can reach conventional statistical significance while having a small standardized effect size, emphasizing why the P-value should not be interpreted in isolation from the magnitude and precision of the effect.

**Figure 4:**
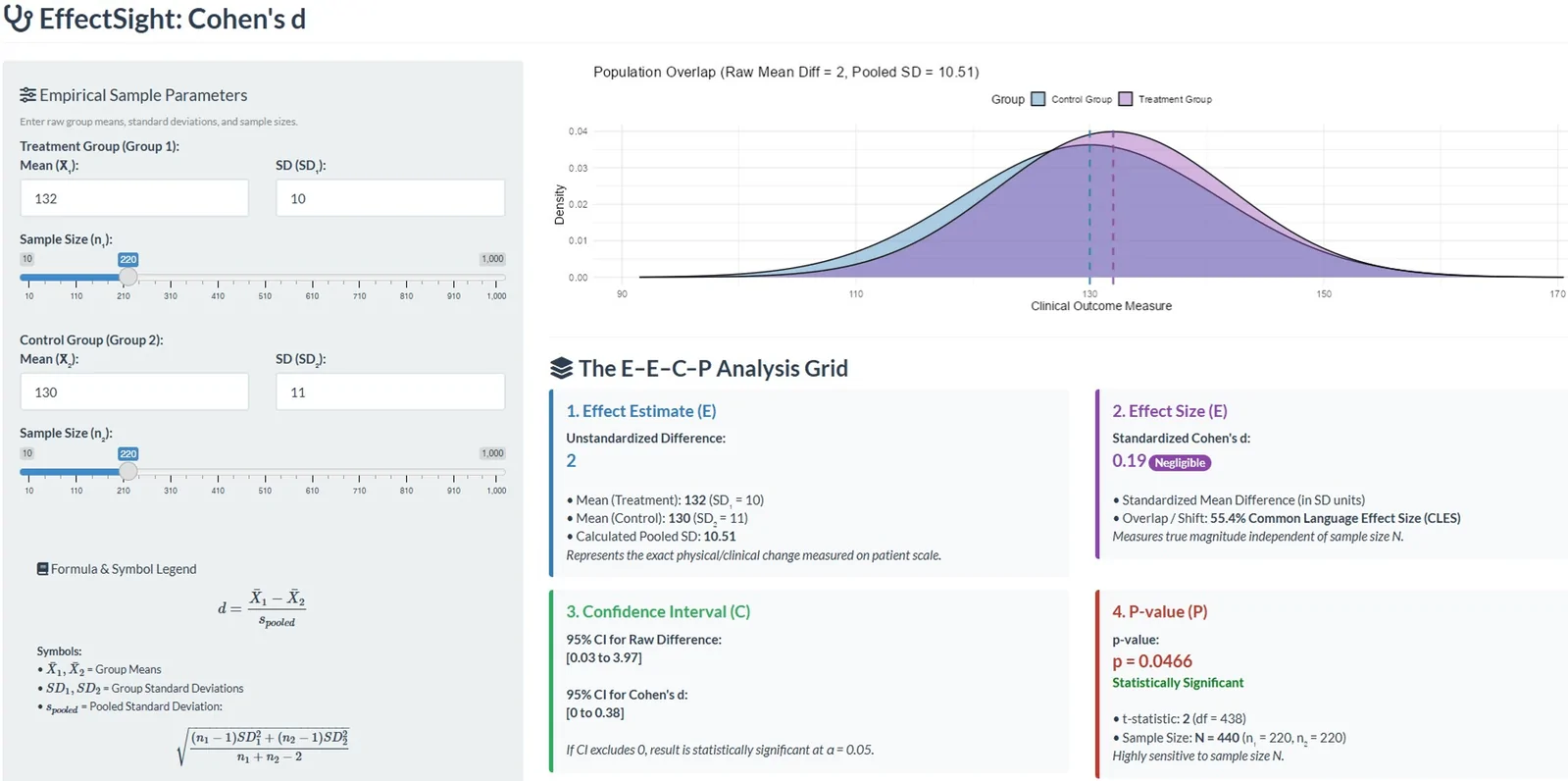
User interface of EffectSight application showing an example of mean difference in effect estimate, effect size, confidence interval, and p-value in E-E-C-P framework This is a screenshot of the Shiny application EffectSight: Cohen's d [15], prepared by Dr. Himel Mondal. Cohen's d was calculated as the difference between the means of the two groups divided by their pooled standard deviation [16]. E: effect estimate/effect size. C: confidence interval. P: p-value.

The app is intended to illustrate the E-E-C-P framework using examples based on the t-test and Cohen’s d. Users can enter different example values to demonstrate the four components of the framework. However, the app is a teaching and visualization tool and should not be considered an empirical validation of the E-E-C-P framework across different statistical methods or research settings.

## Discussion

Interpretation of biomedical findings should not rely on the P-value alone. The E-E-C-P framework, comprising effect estimate, effect size, confidence interval, and P-value, provides complementary information about a result. The effect estimate communicates the observed change in clinically meaningful units, while a standardized effect size describes its magnitude relative to variability. The confidence interval indicates the precision of the estimate, and the P-value indicates the statistical evidence against the null hypothesis. These measures address different questions and should therefore be interpreted together [19].

The distinction between clinical magnitude and statistical significance is particularly important. A small effect may achieve a low P-value in a large sample, whereas a potentially important effect may have a wide confidence interval and a non-significant P-value in a small study [20]. Standardized effect sizes such as Cohen's d facilitate comparison across studies and measurement scales, but clinical interpretation is often better supported by non-standardized measures expressed in meaningful units [21]. Consequently, effect size thresholds should be considered contextual guidelines rather than universal definitions of clinical importance.

The EffectSight Shiny application translates these concepts into an interactive learning environment. By allowing users to modify group means, variability, and sample size, the application demonstrates how effect magnitude, precision, and statistical significance can change independently. This provides a practical pedagogical complement to conventional teaching based on formulas and static examples. EffectSight's application code is available as open-source R code that anyone can review and modify to suit their needs [15]. The application was computationally verified against standard statistical formulas for effect size, pooled standard deviation, and confidence intervals.

Limitation

The EffectSight application was not formally tested for usability, user feedback assessments, or external educational validation. The current implementation is strictly limited in its illustrative scope to two-group comparisons using Cohen’s d under the assumptions of normality. It does not support paired designs, non-parametric distributions, or advanced multivariable analyses. The calculated statistical effect sizes reflect standardized mathematical magnitudes rather than guaranteed clinical relevance, which ultimately requires contextual evaluation against minimal clinically important differences.

## Conclusions

Effect estimate, effect size, confidence interval, and P-value provide distinct and complementary information for interpreting biomedical research. The E-E-C-P framework offers a simple approach for asking what happened, how large the effect was, how precisely it was estimated, and what statistical evidence supports it. The accompanying EffectSight Shiny application provides an accessible, interactive tool for teaching these concepts and reinforcing the principle that statistical significance should not be equated with clinical importance.

## References

[1] Panos GD, Boeckler FM (2023). Statistical analysis in clinical and experimental medical research: simplified guidance for authors and reviewers. Drug Des Devel Ther.

[2] Ahmed ES, Butt MN (2025). The misunderstood P-value: why statistical significance is not enough in clinical practice. Br J Anaesth.

[3] Sharma H (2021). Statistical significance or clinical significance? A researcher's dilemma for appropriate interpretation of research results. Saudi J Anaesth.

[4] Boscardin CK, Sewell JL, Tolsgaard MG, Pusic MV (2024). How to use and report on p-values. Perspect Med Educ.

[5] Lu Y, Belitskaya-Levy I (2015). The debate about p-values. Shanghai Arch Psychiatry.

[6] Higgins JPT, Li T, Deeks JJ (2024). Choosing effect measures and computing estimates of effect. Cochrane Handbook for Systematic Reviews of Interventions, Version 6.5.

[7] Sullivan GM, Feinn R (2012). Using effect size - or why the p value is not enough. J Grad Med Educ.

[8] Hedges LV (2025). Interpretation of the standardized mean difference effect size when distributions are not normal or homoscedastic. Educ Psychol Meas.

[9] O'Brien SF, Yi QL (2016). How do I interpret a confidence interval?. Transfusion.

[10] Nahm FS (2017). What the P values really tell us. Korean J Pain.

[11] Kallogjeri D, Piccirillo JF (2023). A simple guide to effect size measures. JAMA Otolaryngol Head Neck Surg.

[12] Davis SL, Johnson AH, Lynch T (2021). Inclusion of effect size measures and clinical relevance in research papers. Nurs Res.

[13] Ialongo C (2016). Understanding the effect size and its measures. Biochem Med (Zagreb).

[14] (2026). Effect size in research: definition, calculation, reporting, examples. https://www.editage.com/blog/effect-size/.

[15] (2026). EffectSight: a shiny application to visualize Cohen's d. https://figshare.com/articles/software/EffectSight_A_Shiny_application_to_visualize_Cohen_s_d/33319302.

[16] Kim HY (2015). Statistical notes for clinical researchers: effect size. Restor Dent Endod.

[17] The Comprehensive R Archive Network. https://cran.r-project.org (2026). The comprehensive R archive network. https://cran.r-project.org.

[18] (2026). RStudio IDE. https://posit.co/downloads.

[19] Dunkler D, Haller M, Oberbauer R, Heinze G (2020). To test or to estimate? P-values versus effect sizes. Transpl Int.

[20] Gómez-de-Mariscal E, Guerrero V, Sneider A, Jayatilaka H, Phillip JM, Wirtz D, Muñoz-Barrutia A (2021). Use of the p-values as a size-dependent function to address practical differences when analyzing large datasets. Sci Rep.

[21] Collins JP (2019). Measures of clinical meaningfulness and important differences. Phys Ther.

